# Non-Athletic Cohorts Enrolled in Longitudinal Whole-Body Electromyostimulation Trials—An Evidence Map

**DOI:** 10.3390/s24030972

**Published:** 2024-02-02

**Authors:** Miriam Beier, Daniel Schoene, Matthias Kohl, Simon von Stengel, Michael Uder, Wolfgang Kemmler

**Affiliations:** 1 Institute of Radiology, University Hospital Erlangen, 91054 Erlangen, Germany; miriambeier1@gmail.com (M.B.); daniel.schoene@fau.de (D.S.); simon.von.stengel@fau.de (S.v.S.); michael.uder@uk-erlangen.de (M.U.); 2 Department of Medical and Life Sciences, University of Furtwangen, 78056 Villingen-Schwenningen, Germany; matthias.kohl@hfu.eu; 3 Institute of Medical Physics, Friedrich-Alexander University of Erlangen-Nürnberg, 91052 Erlangen, Germany

**Keywords:** whole-body electrostimulation, electromyostimulation, cohorts, function, body composition, diseases, longitudinal studies

## Abstract

Whole-body electromyostimulation (WB-EMS) can be considered as a time-efficient, joint-friendly, and highly customizable training technology that attracts a wide range of users. The present evidence map aims to provide an overview of different non-athletic cohorts addressed in WB-EMS research. Based on a comprehensive systematic search according to PRISMA, eighty-six eligible longitudinal trials were identified that correspond with our eligibility criteria. In summary, WB-EMS research sufficiently covers all adult age categories in males and females. Most cohorts addressed (58%) were predominately or exclusively overweight/obese, and in about 60% of them, diseases or conditions were inclusion criteria for the trials. Cohorts specifically enrolled in WB-EMS trials suffer from cancer/neoplasm (n = 7), obesity (n = 6), diabetes mellitus (n = 5), metabolic syndrome (n = 2), nervous system diseases (n = 2), chronic heart failure (n = 4), stroke (n = 1), peripheral arterial diseases (n = 2), knee arthrosis (n = 1), sarcopenia (n = 3), chronic unspecific low back pain (n = 4), and osteopenia (n = 3). Chronic kidney disease was an eligibility criterion in five WB-EMS trials. Finally, three studies included only critically ill patients, and two further studies considered frailty as an inclusion criterion. Of importance, no adverse effects of the WB-EMS intervention were reported. In summary, the evidence gaps in WB-EMS research were particular evident for cohorts with diseases of the nervous and cerebrovascular system.

## 1. Introduction

Whole-body electromyostimulation (WB-EMS) is a training technology with increasing popularity world-wide. In contrast to the recognized local EMS predominately applied in orthopedic therapy, WB-EMS stimulates most major muscle groups simultaneously but with a dedicated impulse intensity and without the relevant orthopedic demands. Thus, much more than local EMS, WB-EMS can be considered as a time-effective, joint-friendly, and highly customizable alternative to conventional exercise [1]. Whilst this aspect is attractive for athletes looking to improve sport-specific skills, reduce the risk of injuries, or adverse effects (i.e., back pain), the main population for WB-EMS application, however, is sedentary or at least non-athletic adults [2] wanting to increase physical fitness, function, or health-related outcomes. A quick look at the rapidly increasing and very complex research on WB-EMS reveals an unequal addressing of cohorts by present studies. Most of the WB-EMS trials focus on healthy adults, with fewer studies covering participants with specific conditions or diseases. This might be attributable to the rather stringent index of absolute or relative contraindications published by a German expert group in 2019 [3], based in part on an overcautious approach due to a lack of evidence and several adverse effects of intense WB-EMS application reported in the media. In stark contrast, Belt Electrode-Skeletal Muscle Electrical Stimulation (B-SES), a neuromuscular stimulation technique that stimulates large muscle areas and can thus be considered as very closely related to WB-EMS, focuses predominately on frail cohorts in a hospital setting. Adding both systems might increase the evidence for a wider applicability of WB-EMS on different outcomes in varying non-athletic cohorts. Accordingly, in order to provide evidence and identify gaps in knowledge and/or future research on WB-EMS needs [4], we conducted a systematic and comprehensive review of WB-EMS and the eligible B-SES literature. The resulting evidence (gap) map [5] aimed to provide an overview of cohorts enrolled in WB-EMS trials and to support the readjustment of potentially excessive contraindications.

## 2. Methods

The literature search for this systematic review and evidence map followed the Preferred Reporting Items for Systematic Reviews and Meta-Analyses (PRISMA) Statement.

### 2.1. Search

**Figure 1:**
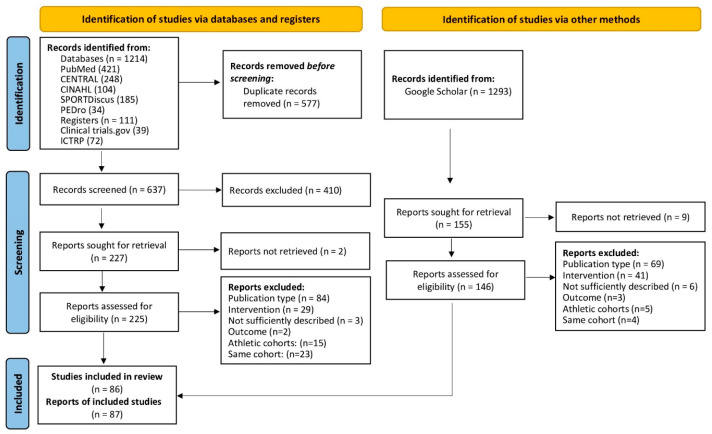
Flow diagram of search process according to PRISMA [102].

Study reports from the five electronic databases (Medline [PubMed], The Cochrane Central Register of Controlled Trials [CENTRAL], Cumulative Index to Nursing & Allied Health [CINAHL via Ebsco Host], SPORTDiscus (via Ebsco Host), and The Physiotherapy Evidence Database [PEDro]), and two study registers (Clinical trial.gov and the WHO’s International Clinical Trials Registry Platform [ICTRP]) published from their incentives up to 6 March 2023 were searched without language restrictions (Figure 1). Strategies were developed applying free-text words as no database-specific key words (e.g., MeSH, Thesaurus) were identified. We piloted our search and found a good balance for maximizing sensitivity and precision by (a) constructing the search around the term whole-body electromyostimulation only and (b) searching the title and abstract fields only in PubMed, CINAHL, and SPORTDiscus, excluding Medline hits in CINAHL and by applying the ‘Trials’ filter in CENTRAL. To identify additional study reports, we used Google Scholar manually on the same date we accessed the medical databases (Figure 1). The full strategies can be found in Supplemental Table S1.

### 2.2. Selection Process

Titles, abstracts, and full texts were independently screened by two reviewers against the pre-specified eligibility criteria. Disagreements were resolved by discussion or with the help of a third reviewer. The reasons for excluding ineligible studies were recorded. In case of missing data or doubtful information, authors were contacted a maximum of three times within a four-week period.

### 2.3. Eligibility Criteria

#### 2.3.1. Study Design

We included all longitudinal study designs except single-case studies. Review articles, editorials, conference abstracts, and letters were also not considered. The same criteria were applied for bachelor’s or master’s theses, while doctoral theses (dissertations) were included.

#### 2.3.2. Population

Sedentary or at least non-athletic cohorts, independently of participant characteristics, were included. Cohorts comprised of athletes or sport students were excluded. However, recreational sports persons were accepted.

#### 2.3.3. Comparators

Type or even presence of a control group was not considered as an eligibility criterion.

#### 2.3.4. Intervention

We only included studies that applied whole-body electromyostimulation (WB-EMS [6]) or other kinds of electromyostimulation able to stimulate large muscle areas (≥50% of skeletal muscle mass) simultaneously (This refers solely to the Belt Electrode-Skeletal Muscle Electrical Stimulation (B-SES) approach that stimulates hip and lower extremity muscle groups). Studies that applied local EMS or focused on single muscle groups were not considered.

#### 2.3.5. Outcomes

In the present analysis, we included studies that focus on physical fitness, function, body composition, and health-related outcomes. Special emphasis was further placed on the safety aspects of the WB-EMS intervention and in particular adverse effects. In detail, an “adverse event” (AE) was defined as any untoward medical occurrence, unintended disease or injury, or any untoward clinical signs, including an abnormal laboratory finding related to the WB-EMS application. However, temporary muscular soreness after WB-EMS application was not considered as an adverse effect. A “serious adverse event” was defined as any adverse effects of the WB-EMS application that led to death or serious deterioration in the health of the subject (e.g., life-threatening illness/injury, permanent impairment of a body structure/body function, hospitalization, chronic disease).

### 2.4. Data Management

Search results were downloaded and imported to Endnote. Duplicates were identified and excluded based on the method proposed by Bramer et al. [7]. Title and abstract screening as well as full-text screening were conducted using Endnote. In cases of multiple publications that addressed an identical cohort but reported varying outcomes (e.g., [8,9,10,11]), only the main publication was included.

### 2.5. Data Items

A Microsoft Excel table, applied in former studies [12,13] and modified for the present research topic, was used to extract relevant data from the included studies. One author extracted the study, participant, and intervention characteristics, and two other authors checked and confirmed the results. The table was structured into several domains. Publication characteristics include information related to the study type, first author, year, and the country of the publication, while study characteristics listed, for example, the number of study arms, sample size in WB-EMS and control group, comparator (i.e., predominately sedentary control group or active control), and methodologic quality of the studies as determined using the Physiotherapy Evidence Database (PEDro) Scale Risk of Bias Tool.

Intervention characteristics include the following: (1) The mode of application, i.e., isolated WB-EMS or WB-EMS with voluntary movements that should not relevantly affect outcomes, versus superimposed WB-EMS or exercise in addition to WB-EMS. (2) A WB-EMS system including the corresponding manufacturers. (3) The duration of the application (in months), training frequency (sessions/week), and length of the session (in min). (4) The details of the impulse protocol, i.e., impulse type (mono/bipolar), impulse frequency (in Hz), impulse breadth (in µs), impulse intensity, impulse application (continuous or intermittent impulse), length of the impulse phase (in s), and (if applicable) intermittent impulse break (in s).

Due to the topic of the present evidence map, special emphasis was placed on cohort and study characteristics. The cohort and participant characteristics include, in particular, gender, age, BMI, baseline training status, conditions and diseases, drop out, adherence to the WB-EMS protocol, and adverse effects. The trials were categorized into studies with predominately healthy cohorts and studies that focused on participants with specific health-related problems, syndromes (e.g., metabolic syndrome), or diseases. Where applicable, study cohorts were classified according their conditions and diseases by applying the International Statistical Classification of Diseases and Related Health Problems (ICD-10-GM, [14]).

### 2.6. Quality Assessment

Eligible studies were assessed for risk of bias by two independent reviewers using PEDro [15], specifically dedicated to physiotherapy and/or exercise studies. In case of inconsistencies, a third independent reviewer made the decision. Studies with >7 score points were classified as high, 5–7 score points were classified as moderate, and <5 score points were classified as low methodological quality studies, respectively [16].

### 2.7. Data Synthesis

**Figure 2:**
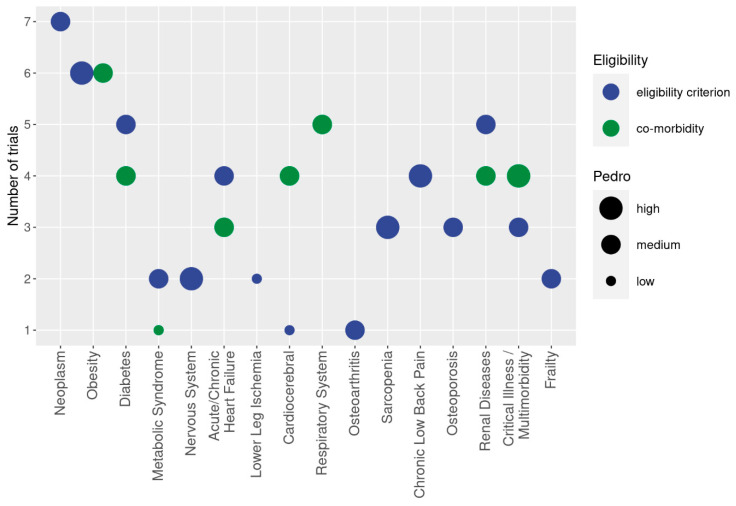
Cohorts with diseases and conditions addressed in WB-EMS trails. The *y*-axis presents the number of studies that focus on the corresponding cohort (*x*-axis). Different colors indicate whether the health status of the cohort was applied as a criterion for inclusion (blue) or reported as a simple co-morbidity (green). The size of the bubble indicates the methodologic quality according to PEDro. The biggest size indicates at least one study of high methodologic quality in the domain. The lowest size of the bubble chart represents at least one study of low methodologic quality in the domain.

**Figure 3:**
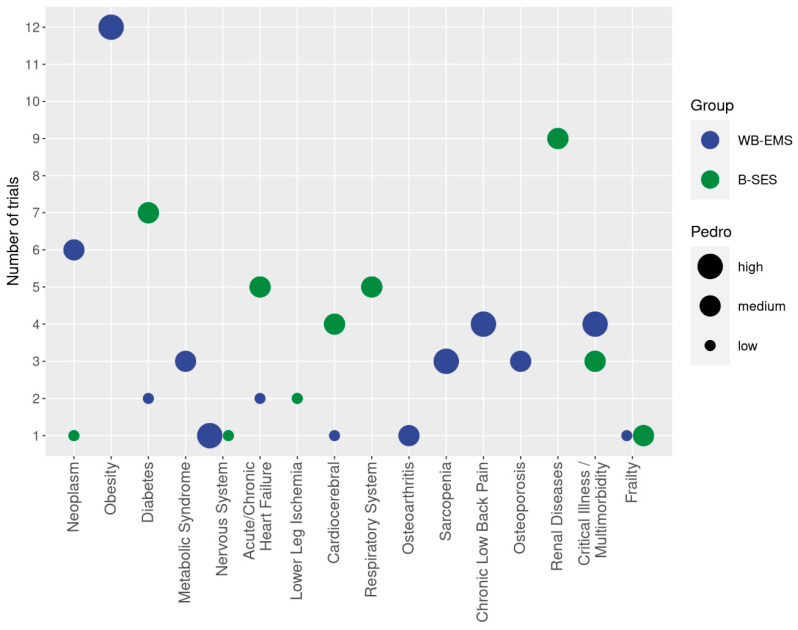
Cohorts with diseases and conditions addressed in WB-EMS trials classified according to WB-EMS (blue) or B-SES (green) application. The *y*-axis presents the number of studies that focus on the corresponding cohort (*x*-axis). The size of the bubble indicates the methodologic quality according to PEDro.

Results are displayed for all studies in tables showing the publication and study characteristics, exercise and stimulation characteristics, and cohort and participant characteristics of the studies included. To provide a rapid overview in the present evidence map, bubble charts with 4 dimensions were created on the *x*-axis, with the health status of participants determined according to ICD-10-GM categories (Figures 2 and 3). The *y*-axis presents the number of studies that focus on the corresponding cohort, while the color of the bubble represents either WB-EMS vs. B-SES application (Figure 3), or the dedicated health status of the cohort applied as a criterion for inclusion or reported as a simple co-morbidity (Figure 2). Finally, the size of the bubble indicates the methodologic quality according to PEDro. The biggest size indicates at least one study of high methodologic quality (i.e., PEDro Score ≥ 8 score points [16]) in the domain. The lowest size of the bubble chart represents at least one study of low methodologic quality.

## 3. Results

Of the 1103 records, 86 longitudinal studies/projects with 87 cohorts were finally included in the present evidence map (Figure 1) [11,17,18,19,20,21,22,23,24,25,26,27,28,29,30,31,32,33,34,35,36,37,38,39,40,41,42,43,44,45,46,47,48,49,50,51,52,53,54,55,56,57,58,59,60,61,62,63,64,65,66,67,68,69,70,71,72,73,74,75,76,77,78,79,80,81,82,83,84,85,86,87,88,89,90,91,92,93,94,95,96,97,98,99,100,101].

### 3.1. Publication and Study Characteristics

Table 1 displays the publication and study characteristics of the included trials. The vast majority of the studies were RCTS (69%). Most of the randomized controlled trials (RCTS, 69%) applied a parallel group design, and three short-term studies provided a cross-over design [42,58,93]. Nineteen non-randomized controlled trials (NRCTs, 22%) and eight (9%) intervention studies without control groups [28,30,34,54,63,88,92,96] were also included. Predominately due to the study design, the methodological quality according to PEDro (Table 1) varies considerably. Considering that NRCTs and in particular intervention studies without control groups failed to obtain points for randomization, allocation concealment, blinding, or even group comparison [15], the study design reflects a low methodological quality according to PEDro. Furthermore, considering that the proper blinding of the participants (i.e., a retrospective query of all participants as to which group they think they belonged to) and particularly the caregivers (WB-EMS instructors) in exercise studies is hardly possible, a score index of eight on the ten-point PEDro scale can be considered as the realistic maximum in WB-EMS studies.

Most studies were conducted in Germany (n = 28), Japan (n = 18), Korea (n = 8), Spain (n = 5), Iran (n = 5), Brazil (n = 4), and Italy (n = 3). The vast majority of studies were published after 2015 (>90%). The number of study arms varied from one [30,34,54,63,88,92,96] to five [62]. The number of participants per study arm varied between three [65] and ninety-six [83] in the WB-EMS group(s), and (if applicable) from three [65] to eighty [56] in the control group(s). The study length varied from 10 days [90] to 12 months [47,98]. Unfortunately, 15 studies (16 subgroups) failed to report the drop-out rate and did not respond to our queries or were unable to calculate the drop-out rate retrospectively. The drop-out rate of the remaining studies varied from 0% to 59%. Of the eleven studies with drop-out rates ≥25%, nine studies focused on patients with severe complaints and diseases (e.g., end-stage kidney disease, stroke, critically illness, cancer) [37,39,42,51,60,80,81,90] (Table 1).

### 3.2. Exercise and WB-EMS Characteristics

Supplemental Table S2 displays the exercise and WB-EMS characteristics of the studies. Sixty-nine studies applied whole-body electromyostimulation (WB-EMS), and eighteen studies applied Belt Electrode-Skeletal Muscle Electrical Stimulation (B-SES). About two thirds of the WB-EMS studies used miha-bodytec devices (Gersthofen, Germany) while the B-SES technique exclusively used HomerIon (Tokyo, Japan). Although difficult to classify, about 80% of the cohorts conducted predominately isolated WB-EMS, i.e., a protocol without adjuvant or additional movements with relevant effects on the primary study outcome. The remaining studies applied either superimposed WB-EMS (i.e., exercises intensified by WB-EMS) or combined WB-EMS and conventional exercise. In parallel, three quarters of the studies provided an active WB-EMS mode, i.e., predominately movements during the impulse phase. B-SES studies generally focused on a passive EMS application mode. The WB-EMS training frequency varied from daily application [60,61,65] to one session per week [21,46,56,59,79,86,94,95,99,101]. The average training frequency of the B-SES studies was significantly (*p* < 0.001) higher compared with the WB-EMS studies (4.1 ± 1.7 versus 2.0 ± 0.8 sessions/week). The session length varied between 12 and 20 min [21] and 90 min [17]. Most studies (n = 75) applied WB-EMS or B-SES protocols of 20–30 min (Table 2). All but one study [53] focused on low-frequency stimulation protocols from 4 Hz [42] to 100 Hz [87] and impulse widths of 200–400 µs. The majority of studies applied intermittent WB-EMS protocols, predominately with 4–6 s of impulse and 2–4 s of impulse break; only 4 studies provided a consistently continuous impulse during the session [33,42,57,93] (Table 2). At least seventeen studies [17,20,21,22,23,31,43,44,62,66,67,68,70,75,77,79,87] worked with variable WB-EMS programs, i.e., they applied varying WB-EMS parameters, predominately including impulse frequency, width, or impulse phase/break, during the session or during the intervention. Apart from a few studies that solely evaluated the effects of low impulse intensity [40,46,57] and one study that applied very high impulse intensity [92], all other WB-EMS studies scheduled moderate to high impulse intensities based on the Borg CR-10 (…or rarely CR-20) scale, rate of the maximum impulse tolerance (60–80% 1 MT), or according to the authors’ estimation. In contrast, several B-SES studies applied stimulation protocols up to the maximum tolerable intensity (e.g., [27,36,42,65,93]).

Apart from the WB-EMS application, some studies applied specific diets (e.g., [18,23,35]) or provided protein supplements [11,46,72,100].

### 3.3. Participant and Cohort Characteristics

Table 2 reports characteristics of the cohorts and study participants. In summary, the studies cover all (adult) age categories in female, male, and mixed gender categories. Most studies (51%) included men and women, 33% focused on female participants, and 16% focused on male participants. About 20% of the studies addressed cohorts largely independently of age. Eleven studies (12%) focused exclusively on cohorts 30 years and younger, and twelve studies (14%) included only participants 70 years and older. With respect to premenopausal women, no longitudinal study focused on issues related to pregnancy, puerperium, or lactation.

Forty-six of the seventy-eight trials that reported corresponding data addressed cohorts that were predominantly or exclusively overweight (i.e., mean BMI ≥ 25.0 kg/m^2^). However, only about one third of them defined overweight or obesity as a criterion for inclusion.

Diseases or conditions were criteria for inclusion in 60% of the WB-EMS/B-SES studies. Apart from two studies [27,57] that focused on healthy young (22 ± 2) or older people (60–90 years) with limited mobility, all the other B-SES studies addressed predominantly hospitalized people with severe diseases. In contrast, about half of the WB-EMS trials addressed apparently healthy cohorts; further, only one WB-EMS study [91] applied an ambulatory setting. A large variety of conditions and diseases were reported; thus, following the ICD 10 classification [14], the cohorts were categorized into different domains and subcategories (Figure 2 and Figure 3). Due to the critically ill and/or multi-morbid status of some cohorts, the corresponding trials were cited for more than one classification.

### 3.4. Neoplasms

In summary, six studies with seven study groups [36,76,80,81,82,83] addressed cohorts with malignant neoplasms. In particular, the research group of Zopf et al. [76,80,81,82,83] focused on this issue, applying WB-EMS for 12 weeks each. So far, the authors have published data on their ongoing advanced cancer project [80] with subgroup analyses on hematological malignancies [81], gastro-intestinal [76], pancreatic [83], prostate, and colorectal cancer [82]. Hamada et al. [36] focused on patients at the early stage of an allogeneic stem cell transplant, predominately in people with acute leukemia applying B-SES for four post-transplantation weeks (A further B-SES case–control study [103] not included in this evidence map focused on the same cohort). Other studies did not focus on but also included cancer patients [88]. Of importance, none of the studies reported adverse effects during the intervention. Evidence for WB-EMS or B-SES application in cancer patients provided by the non-randomized studies and subgroup analysis can be considered moderate (evidence level IIa).

### 3.5. Endocrine, Nutritional and Metabolic Diseases

A large number of studies focused on cohorts with metabolic disorders and diseases. Apart from two studies with sarcopenic obesity cohorts [11,46], ten further studies addressed cohorts with obesity [17,22,23,38,48,74,75,91,96,100]. However, only six studies considered “obesity” as an eligibility criterion [11,22,46,48,74,75] (Song et al. [85] described his cohort of female students as “obese”; but due to BMI (26.1 kg/m^2^) or body fat rate (28% as determined by BIA), this cohort can be considered as overweight only. However, this error can be attributable to the translation (Korean–English)). One further study applied abdominal obesity [47] as an eligibility criterion. Apart from one exception with overweight participants [88], all trials on B-SES conducted exercises with participants with a normal BMI or even with severely underweight particpants [42]. Of importance, none of the studies on obesity reported adverse effects during the intervention. Considering the evidence level of the studies, with three RCTs [11,22,75] of high methodologic quality that applied obesity as a criterion for inclusion, the evidence level provided for the EMS application can be classified as high.

Cohorts with non-insulin-dependent diabetes mellitus (NIDDM) were addressed in five randomized and non-randomized trials or intervention studies without CG [38,39,89,93,96] that applied WB-EMS (n = 2) or B-SES (n = 3) for two to four months. Two of the B-SES studies included hospitalized cohorts with diabetic ulcers undergoing minor amputation [39] or end-stage kidney disease [93]. Additionally, four other B-SES studies did not focus on but included a large proportion of participants with diabetes [37,54,55,88]. Of importance, a further three moderate to high quality RCTs [23,43,74] focused on cohorts with metabolic syndrome and applied WB-EMS for 3–6 months. Unfortunately, one [93] study on NIDDM and MetS cohorts failed to report adverse effects. Summarizing the evidence of the studies, with two moderate methodologic quality RCTs [39,93], evidence for EMS application in NIDDM cohorts can be considered moderate–high. Additionally, three low-moderate quality RCTs that applied MetS as a criterion for inclusion [43,74] and did not observe adverse effects might increase the evidence for WB-EMS application in people with cardiometabolic diseases.

### 3.6. Diseases of the Nervous System

Only a few studies focused on cohorts with diseases of the nervous system [29,58]. While the high-quality RCT of Di Cagno et al. [29] focused on stage 1 (mild)–3 (moderate) Parkinson’s disease in patients 50–80 years old for their 12-week WB-EMS trial, the NRT of Mori et al. [58] addressed Huntington’s disease patients during dialysis with B-SES for 6 weeks (Another case–control study [104] not included in the evidence map focused on B-SES and virtual reality-guided balance training (30 days) for managing paraplegia after spinal cord infarction). While DiCagno et al. [29] observed no adverse effects, unfortunately, Mori et al. [29] did not report the unintended effects of B-SES application.

### 3.7. Cardiovascular Diseases

The non-controlled cohort 2.5-month WB-EMS study of Fritsche et al. [34] and the 4-month NRCT of van Buuren [97] solely included participants with chronic heart failure [34,97]. Two other moderate quality B-SES studies [55,90] selected acute heart failure as an eligibility criterion and applied 10 and 14 days of B-SES during hospitalization. In parallel, about 50% of the critically ill patients included in the two low and moderate methodologic quality RCTs of Nakamura et al. [60,61], and 70% of the older hemodialysis patients included in the moderate quality RCT of Homma et al. [37], displayed heart failure, cardiopulmonary arrest [60,61], or had a history of ischemic heart disease [37]. Apart from two studies [60,61] with critically ill patients that failed to report unintended side effects related to the intervention, none of the studies reported adverse effects.

Severe ischemia of the lower limbs/peripheral arterial diseases [54,65] was an eligibility criterion in two low methodologic quality B-SES trials. Neither study observed adverse effects related to the intervention.

The low methodologic quality RCT of Lukashevich et al. [53] exclusively addressed patients <6 months after a stroke event with high-frequency WB-EMS for 3 weeks. In parallel, the vast majority (16 of 18) of the bedridden older participants of the moderate quality RCT of Kataoka et al. [42] suffered from cerebral infarction, cerebral or subarachnoid hemorrhage, or hypoxic ischemic encephalopathy (B-SES). Among the two B-SES studies of Nakamura et al. [60,61] with critically ill patients, and the moderate quality B-SES RCT of Homma et al. [37], about half of the patients suffered from stroke [37] or displayed a history of cerebrovascular events/disease. Apart from the two low to moderate quality RCTs of Nakamura et al. [60,61], with their particularly vulnerable cohort that did not report adverse effects, none of the other studies that focused on “stroke patients” reported adverse effects of the EMS intervention.

Surprisingly, hypertonic cohorts were not specifically addressed by longitudinal studies. However, the proportion of study participants with hypertension averaged >50 to >90% in four B-SES trials [37,55,88,90]. None of the four low to moderate quality studies reported adverse effects of the intervention. Nevertheless, due to the high incidence of hypertension in the adult population, a dedicated study that provides evidence for the safe application of WB-EMS in this cohort would be quite welcome.

### 3.8. Diseases of the Respiratory System

No study has so far applied diseases of the respiratory system as a criterion for inclusion in WB-EMS studies. However, 30% and 60% of the patients in the two low and moderate quality B-SES RCTs on critically ill patients of Nakamura et al. [60,61] suffered from respiratory failure. In parallel, three other low-moderate methodologic quality studies reported the inclusion of patients with respiratory failure or COPD [55,64,90]. While the latter three studies did not observe unintended side effects, Nakamura et al. [60,61] did not report adverse effects in his critically ill patients.

### 3.9. Musculoskeletal and Connective Tissue Diseases

So far, only one moderate quality WB-EMS RCT of 8 weeks applied (knee) osteoarthritis as the main criterion for inclusion [70]. The study reported no adverse effect of the WB-EMS application.

Sarcopenia, recently included in the ICD 10 GM (M62. 84), was specifically addressed by two high-quality WB-EMS RCTs of 4 and 6 months in an ambulatory setting [11,46], and by one 4-week RCT conducted in a stationary setting [91]. In summary, none of the studies observed adverse effects of the EMS protocol. Considering the poor muscle mass or/and function of patients reported by many B-SES studies (e.g., [37,42,60,61,63,64,90,93]) a large proportion of these cohorts might also suffer from sarcopenia.

Non-specific chronic low back pain was the primary eligibility criterion in two 3-month high-quality WB-EMS studies and two 6- and 8-week NRCTs [51,56,84,99]. None of the trials reported adverse effects during the intervention.

Osteopenia or osteoporosis was the main criterion for inclusion in three moderate or high-quality WB-EMS studies of 10, 14, and 52 weeks [44,94,98]. None of the studies observed adverse effects of the EMS intervention.

In parallel to sarcopenia, the vast majority of B-SES studies and WB-EMS studies with older people (i.e., 60 years and older) might also include a high proportion of people with osteopenia/osteoporosis; this relates in particular to female cohorts with increased peri- and (early) post-menopausal bone loss [105]. The fact that adverse effects were not observed underscores the safety of EMS application in these older cohorts.

### 3.10. Diseases of the Genitourinary System

Several 6–12-week low to moderate methodologic quality studies applied B-SES during dialysis in patients with chronic kidney diseases [37,58,63,88,93]. At least two other low to moderate quality B-SES studies included a moderate–large proportion of patients with chronic renal disease [55,90] or post renal replacement therapy [60,61]. Unfortunately, four studies that included patients with renal diseases failed to list adverse effects, while the remaining studies did not observe unintended side effects of the EMS intervention.

### 3.11. Critical Illness, Multi-Morbidity

Three low to moderate B-SES studies focused on critically ill patients treated in intensive care units [60,61,64] for 10–40 days. While Nakamura et al. [61] did not address this issue, Nonoyama et al. [64] and Nakamura et al. [60] reported no adverse effects of B-SES application in their study.

When defining multi-morbidity as the simultaneous presence of three or more chronic diseases [106], many B-SES studies and at least four WB-EMS studies [11,25,46,91] included multi-morbid cohorts and applied WB-EMS for one to six months. Although not all B-SES studies focused on this issue, no study reported adverse effects of the EMS application.

### 3.12. Frailty, Functional Limitation

In their moderate quality RCT, Kataoka et al. [42] focused on severely frail, bedridden elderly patients in their 12-week B-SES study. Another two-month low quality WB-EMS pilot study [25] applied frailty as a criterion for inclusion. Boutry-Regard et al. [27] only included older people with limited mobility (…however, the cut-off value for gait speed of 1.5 m/s is considerably above the 0.8 to 1.0 m/s criteria for slow gait speed, e.g., suggested for sarcopenia diagnosis [107,108,109,110]) in their 12-week moderate quality B-SES RCT. None of the studies listed above reported adverse effects of the EMS application.

Apart from these trials, several other studies that focused on critically ill patients [60,61,64], sarcopenia [11,46,91], or end stage kidney disease [93] included a large proportion of frail or physically limited older people. The fact that none of the studies reported unintended side effects might increase the evidence for WB-EMS application in this domain.

### 3.13. Adverse Effects

Apart from ten studies (WB-EMS n = 6; B-SES n = 4), with three studies [58,61,93] addressing cohorts with conditions and diseases, all other studies reported or submitted the prevalence of adverse effects on request. Besides one study [92], and independently of the cohorts addressed, no study reported side effects of the EMS intervention with WB-EMS or B-SES. The only study that reported acute adverse effects of WB-EMS [92] focused on the effects of very high impulse intensities in novice WB-EMS applicants with rhabdomyolysis effects in a closely medically supervised setting. In summary, the study reported exceptionally high creatine kinase and myoglobin levels 3–4 days after a one-off 20 min WB-EMS application. However, this cannot be considered as an adverse effect, but as the primary study outcome.

## 4. Discussion

This project aimed to identify and summarize studies that reported data on longitudinal WB-EMS application or closely related techniques able to stimulate large muscle areas in different non-athletic adult cohorts. In summary, the present evidence map provided evidence for the (safe) application of WB-EMS (including B-SES) techniques in several, even critically ill, cohorts covered by the 86 studies included.

With respect to age and gender, most cohorts were addressed by the trials. This particularly includes older women and men who are either institutionalized, hospitalized, or living in community living centers, and who are specifically relevant for joint-friendly, highly customizable, and consistently supervised training technologies. Although WB-EMS-induced reductions of total or regional body fat are limited [111], many studies focused on overweight cohorts. Few of the studies applied a combination of WB-EMS and diet [18,23,35,100]. Although some specific research questions remain, we feel that evidence for WB-EMS application in overweight cohorts is sufficiently provided.

Apart from cohorts with overweight or obese patients, the majority of trials with sedentary, non-athletic adults addressed cohorts with health-related problems and limitations. This refers in particular to B-SES, which is used primarily in hospitals and care facilities. It is of crucial importance that no study, whether it involved advanced cancer, diabetes, stroke, Parkinson’s disease, chronic heart failure, pAVK, COPD, sarcopenia, psteoporosis, pre-frailty or frailty, chronic renal failure, or even critically ill cohorts, observed adverse effects related to WB-EMS or B-SES application. However, one should bear in mind that the studies provided close supervision predominately by medical staff. Summarizing the results of the evidence map for health issues, a sufficient body of evidence for WB-EMS application is available for cohorts with (1) non-specific chronic low back pain, (2) sarcopenia, (3) osteopenia/osteoporosis, (4) obesity, (5) non-insulin-dependent diabetes mellitus and MetS, (6) cancer/neoplasms, (7) chronic renal diseases, (8) multi-morbidity, and (9) critically ill hospitalized patients, although for the latter group, adverse effects were not consistently provided.

Still, insufficient evidence is available for WB-EMS application in cohorts with (1) acute or chronic heart failure, (2) diseases of the respiratory system, or (3) cerebrovascular diseases, a condition particularly promising for WB-EMS due to the lack of other training options.

Our study identified several gaps of WB-EMS research with respect to the cohorts addressed. However, not all health-related domains are equally relevant for dedicated WB-EMS research. This applies in particular for people with local limitations (e.g., arthropathies, spondylopathies) accessible for local EMS-application. On the other hand, cohorts with conditions or diseases that benefit from the simultaneous stimulation of large muscle groups and limited options for conventional exercise training will be particularly important for WB-EMS research. The corresponding gaps in WB-EMS research concerning cohorts with Alzheimer’s diseases, polyneuropathies, myoneural disorders, or multiple sclerosis, but also with stroke or general immobilization, should be addressed with particular emphasis.

Apart from providing evidence for WB-EMS application in varying study cohorts and drawing attention to gaps in the WB-EMS literature, another aim of the present evidence map was to support the considerations of decision makers with respect to future recommendations on absolute and relative contraindications for WB-EMS application. To our best knowledge, only one available publication summarized contraindications for WB-EMS [3]. Although these recommended contraindications focus on the non-medical, commercial German WB-EMS market, most other providers and many researchers consider these recommendations to be mandatory. Briefly addressing the history of these contraindications, the commercial WB-EMS market suffered from a series of adverse effects that resulted in critical discussions in the media and led to a temporary ban in Israel (review in [112]). The lack of mandatory regulations for qualifications for providers and (in particular) instructors led the German expert group on WB-EMS [112] to issue very cautious recommendations in 2019. Meanwhile, a rather dense network of Federal regulations addressed WB-EMS applications (e.g., [113]), which includes the mandatory licensing of WB-EMS instructors/caregivers (e.g., Germany: [114]). Apart from federal regulations, the introduction of “medical WB-EMS”, defined as (1) primarily a therapeutic intervention, (2) based on an existing diagnosis, (3) provided by qualified medical–therapeutic personnel, (4) in compliance with current guidelines, and (5) using medical devices [112], allows for the opening of WB-EMS-applications for previously excluded cohorts [3]. We feel that the present evidence map will be helpful in the elaboration of an updated list of relative and absolute contraindications on WB-EMS-application. However, this approach must be conducted in close liaison with expert groups.

Some features of this evidence map might be irritating or hard to grasp for the reader. First of all, the present evidence map focuses on “cohorts” included by WB-EMS studies and thus differs from most evidence maps that address “study outcomes” (e.g., [115]). Both parameters are similarly important, but since a comprehensive analysis and description of both aspects failed, we decided to give priority to the “cohort aspect”. This reflects our aim to provide timely data for the readjustment of absolute and relative contraindication on WB-EMS.

One may argue that combining WB-EMS, defined as the “simultaneous application of electric stimuli via at least six current channels or participation of all major muscle groups with a current impulse effective to trigger muscular adaptations” [6], with the B-SES technique might not be reliable. While many features are comparable (Supplemental Table S2), B-SES neuromuscular stimulation uses a monophasic, exponentially climbing pulse. Further, depending of the B-SES device, five (e.g., [37]) or six (e.g., [36,42]) electrodes are fixed at the waist/lower back and/or thigh and ankles, resulting in a lower stimulation area compared to WB-EMS. Additionally, in contrast to the usual WB-EMS application in an upright standing position, all included trials applied B-SES in a sitting [27] or a (mainly) supine position, predominately in a passive mode, i.e., without voluntary movements during the impulse phase. While the duration of the WB-EMS or B-SES sessions are largely similar, the training frequency of B-SES is significantly higher. The stimulus intensity of B-SES was consistently described as the maximum tolerable impulse intensity without pain (or discomfort); i.e., largely in line with the specification applied by WB-EMS. For both methods, acute stimulation effects on deeper muscle layers of the thigh and lower legs were reported [116,117]. We based our criteria of “safety” on missing adverse effects. We agree with the objection that this did not necessarily indicate that WB-EMS is a harmless exercise technology for every cohort. This particularly refers to applications that were too intense for novice users, which have resulted in severe rhabdomyolysis [92,118]. However, considering recently updated guidelines [119] and the rather restrictive contraindications on WB-EMS [3], we conclude that the safety standards for WB-EMS application are exceptionally high, at least compared with other types of exercise or exercise technologies. Nevertheless, no WB-EMS study exceeded the length of 12 months [98] (and thus long-term adverse effects were not recorded), which indicates the need for the scientific long-term monitoring of WB-EMS application. Further, although not addressed by the present work but nevertheless important for increasing safety, more research on the customization of WB-EMS protocols is required to meet the specific demands, particularities, and preferences of different populations.

Due to the large number of studies, poor information provided, difficulties in proper translation, and partially missing author responses, we might have failed to identify all eligible articles, or always correctly classify or describe the included articles. This may also be attributable to the approach of including all kinds of longitudinal (full-text) studies irrespective of their design. We agree that this would be a limitation when addressing “study outcomes”; however, when addressing “cohorts”, the study design might be of lesser relevance. Nevertheless, it is important to classify the contribution of the single studies for evidence and relevance of the domain. This was covered by considering whether the corresponding trial considered the dedicated disease/condition as a criterion for inclusion or as a simple co-morbidity. In parallel, methodologic quality was rated by the PEDro scale, which is specifically dedicated to clinical physiotherapy and exercise studies. However, this score is not perfectly suitable for non-randomized controlled trials; nevertheless, our approach allows for a rough overview of this important aspect.

Finally, a relevant limitation of the present review is the missing data, which are particularly important in the domain of adverse effects related to WB-EMS application. Although we contacted the corresponding authors several times by email or phone, we failed to obtain data of 10 studies. Unfortunately, these included particularly important studies with vulnerable cohorts [58,61,93]. Apart from the failure of several trails to report adverse effects at all, considering the scarce data provided by all other studies, the monitoring and reporting of adverse effects seem to be a neglected domain in clinical WB-EMS trials overall.

Finally, and of minor importance for the presence study, albeit relevant for studies that focus on effects, many researchers do not report the WB-EMS intervention comprehensibly or completely.

## 5. Conclusions

The present work provides evidence for the application of WB-EMS techniques in a wide range of human cohorts. We conclude that priority should be given to WB-EMS research in people with neurological and cerebrovascular diseases to address existing evidence gaps. This does not exclude advanced research on cohorts repeatedly addressed by WB-EMS studies, however. Nevertheless, the unique selling points of WB-EMS, i.e., its ability to involuntarily stimulate large muscle groups simultaneously with adequate intensity but low orthopedic stress, should be considered in when making decisions about WB-EMS application in eligible cohorts. Another demand related to WB-EMS application in vulnerable cohorts is that ongoing or at least long-running projects should address the long-term safety of WB-EMS. Addressing the safety of WB-EMS applications, although a few articles failed to report adverse effects, none of the identified trials, whether they were conducted with advanced cancer, diabetes, stroke, Parkinson, chronic heart failure, pAVK, COPD, sarcopenia, osteoporosis, frailty, chronic renal failure, or even critically ill cohorts, observed adverse effects related to WB-EMS or B-SES application. Although this of course did not indicate the complete harmlessness of WB-EMS, advanced federal regulations and mandatory qualifications and education for WB-EMS providers and trainers suggest that an easing of the very restrictive contraindications of WB-EMS, at least in consistently supervised settings, should be considered in the near future.

## Figures and Tables

**Table 1:** Publication and study characteristics of the included studies.

	Author	Year	Country	Study- Design	Study Arms (n)	Total Sample Size (n)	Active Control	Methodological Quality
1	Afsharnezhad et al. [17]	2022	IRN	RCT	3	36	yes	low
2	Akcay et al. [18]	2022	TUR	RCT	2	104	yes	moderate
3	Almada et al. [19]	2016	ESP	RCT	2	10	yes	low
4	Amaro-Gahete et al. [21]	2018	ESP	RCT	2	12	yes	moderate
5	Amaro-Gahete et al. [20]	2019	ESP	RCT	4	89	no	moderate
6	Andre et al. [22]	2021	BRA	RCT	2	39	no	high
7	Bellia et al. [23]	2020	ITA	RCT	2	25	no	low
8	Berger et al. [24]	2020	GER	RCT	3	51	no	moderate
9	Blöckl et al. [25] ^a^	2022	GER	NRCT	2^a^	18	yes	low
10	Bostan et al. [26]	2022	TUR	RCT	2	128	yes	low
11	Bouty-Regard et al. [27]	2020	JPN	RCT	3	41	yes	moderate
12	Cetin et al. et al. [28]	2017	TUR	IS without CG-	3	24	n.a.	low
13	DiCagno et al. [29]	2023	ITA	RCT	3	24	no	high
14	Dyaksa et al. [30]	2022	IDN	IS without CG-	1	10	n.a.	low
15	Ethem et al. [31]	2019	IND	RCT	2	18	no	low
16	Evangelista et al. [33]	2019	BRA	RCT	3	58	no	low
17	Evangelista et al. [32]	2021	BRA	RCT	2	30	yes	moderate
18	Fritzsche et al. [34]	2010	GER	IS without CG-	1	15	n.a.	low
19	Ghannadi et al. [35]	2022	IRN	RCT	2	40	yes	low
20	Hamada et al. [36]	2023	JPN	NRCT	2	43	yes	low
21	Homma et al. [37]	2022	JPN	RCT	2	27	no	moderate
22	Houdjijk et al. [38]	2022	NL	NRCT	4	75	yes	low
23	Imaoka et al. [39]	2022	JPN	RCT	2	49	yes	moderate
24	Jee et al. [40]	2019	KOR	RCT	4	54	no	moderate
25	Junger et al. [41]	2020	SVKi	NRCT	2	86	yes	low
26	Kataoka et al. [42]	2019	JPN	RCT	2	16	yes	moderate
27	Kemmler et al. [44]	2010	GER	RCT	2	30	yes	moderate
28	Kemmler et al. [43]	2012	GER	RCT	2	28	yes	moderate
29	Kemmler et al. [47]	2013	GER	RCT	2	46	yes	moderate
30	Kemmler et al. [45]	2016	GER	RCT	2	46	yes	moderate
31	Kemmler et al. [46]	2016	GER	RCT	3	75	no	high
32	Kemmler et al. [11]	2017	GER	RCT	3	100	no	high
33	Kim et al. [48]	2020	KOR	RCT	2	25	yes	moderate
34	Kim et al. [49]	2021	KOR	RCT	4	54	no	moderate
35	Kiriscioglu et al. [50]	2019	TUR	NRCT	2	41	no	low
36	Konrad et al. [51]	2020	GER	NRCT	2	128	yes	low
37	Ludwig et al. [52]	2019	GER	RCT	3	58	no	low
38	Lukashevich et al. [53]	2020	BLR	RCT	3	52	no	low
39	Matsumoto et al. [54]	2020	JPN	IS without CG-	1	4	n.a.	low
40	Matsuo et al. [55]	2022	JPN	NRCT	2	90	yes	low
41	Micke et al. [56]	2021	GER	RCT	3	240	yes	high
42	Miyamoto et al. [57]	2016	JPN	RCT	2	19	no	moderate
43	Mori 2020 et al. [58]	2020	JPN	NRCT	1	14	n.a.	low
44	Müllerova et al. [59]	2022	CZE	RCT	2	21	yes	low
45	Nakamura et al. [60]	2019	JPN	RCT	2	94	yes	low
46	Nakamura et al. [61]	2021	JPN	RCT	2	68	no	moderate
47	Nejad et al. [62]	2021	IRN	RCT	5	40	no	low
48	Noguchi et al. [63]	2017	JPN	IS without CG-	1	8	n.a.	low
49	Nonoyama et al. [64]	2022	JPN	NRCT	2	42	n.a	low
50	Ochiai et al. [65]	2018	JPN	NRCT	2	6	yes	low
51	Özdal et al. [67]	2016	TUR	RCT	2	40	yes	low
52	Öktem et al. [66]	2022	TUR	RCT	2	20	no	low
53	Pano-Rodriguez et al. [68]	2020	ESP	RCT	2	34	yes	moderate
54	Park et al. [71]	2021	KOR	RCT	2	34	no	high
55	Park et al. [70]	2021	KOR	RCT	3	81	no	moderate
56	Park et al. [69]	2021	KOR	RCT	2	24	no	moderate
57	Park et al. [72]	2023	KOR	RCT	4	60	yes	moderate
58	Qin et al. [73]	2022	CHN	RCT	2	25	yes	moderate
59	Reljic et al. [74]	2022	GER	RCT	4	103	no	moderate
60	Ricci et al. [75]	2020	BRA	RCT	2	20	no	high
61	Richter et al. [76]	2019	GER	NRCT	2	75	no	low
62	Sadeghipour et al. [78]	2021	IRN	RCT	3	30	no	moderate
63	Sadeghipour et al. [77]	2022	IRN	RCT	3	45	no	low
64	Sanchez-Infante et al. [79]	2020	ESP	RCT	2	28	yes	moderate
65	Schink et al. [80]	2018	GER	NRCT	2	131	no	low
66	Schink et al. [81]	2018	GER	NRCT	2	31	no	low
67	Schwappacher et al. [82]	2020	GER	NRCT	2	18	no	low
68	Schwappacher et al. [82]	2020	GER	NRCT	2	12	no	low
69	Schwappacher et al. [83]	2021	GER	NRCT	2	12	no	low
70	Silvestri et al. [84]	2023	ITA	NRCT	2	52	yes	low
71	Song et al.et al. [85]	2020	KOR	NRCT	3	30	yes	low
72	Stephan et al. [86]	2023	GER	RCT	2	60	yes	moderate
73	Struhar et al. [87]	2019	CZE	NRCT	3	28	no	low
74	Suzuki et al. [88]	2018	JPN	RCT	2	29	no	low
75	Suzuki. et al. [89]	2018	JPN	IS without CG-	1	12	2	low
76	Tanaka et al. [90]	2022	JPN	RCT	2	39	no	moderate
77	Teschler et al. [92]	2016	GER	IS without CG-	1	11	n.a.	low
78	Teschler et al. [91]	2021	GER	RCT	3	134	no	moderate
79	Tsurumi et al. [93]	2022	JPN	RCT	2	22	no	moderate
80	Vacoulikova et al. [95]	2021	CZE	RCT	3	21	no	low
81	Vacoulikova et al. [94]	2021	CZE	RCT	3	63	yes	low
82	van Buuren et al. [97]	2014	GER	NRCT	3	59	no	low
83	van Buuren et al. [96]	2015	GER	IS without CG-	1	15	n.a.	low
84	von Stengel et al. [98]	2015	GER	RCT	2	76	yes	moderate
85	Weissenfels et al. [99]	2018	GER	RCT	2	30	no	high
86	Willert et al. [100]	2019	GER	RCT	3	90	no	moderate
87	Zink et al. [101]	2021	GER	RCT	2	54	no	moderate

CG: control group; IS: Intervention study; n.a.: not applicable; NRCT: non-randomized controlled trial; RCT: randomized controlled trial; ^a^ Blöckl et al.: older cohorts.

**Table 2:** Cohort and participant characteristics of the included studies.

	Author	Year	Gender	Age (Years)	BMI (kg/m^2^) ^1^	Training-status ^2^	Diseases	Drop-Out (%) ^3^	Adherence (%)	Adverse Effects
1	Afsharnezhad et al. [17]	2022	w	29 ± 3	34.6	well	yes	n.g.	n.g.	n.g.
2	Akcay et al. [18]	2022	m + w	33 ± 1	27.2	moderate	no	0	90	no
3	Almada et al. [19]	2016	m	23 ± 3	23.7	moderate	no	0	n.g.	n.g.
4	Amaro-Gahete et al. [21]	2018	m	27 ± 7	23.8	well	no	14	96	no
5	Amaro-Gahete et al. [20]	2019	m + w	53 ± 5	26.8	untrained	no	17	99	no
6	Andre et al. [22]	2021	m + w	39 ± 2	40.5	untrained	yes	15	91	no
7	Bellia et al. [23]	2020	m + w	49 ± 7	40.1	moderate	yes	23	90	no
8	Berger et al. [24]	2020	m + w	26 ± 3	23.8	moderate	no	12	100	no
9	Blöckl et al. [25]	2022	m + w	80 ± 4	26.2 ^4^	untrained	yes	14	88	no
10	Bostan et al. [26]	2022	m + w	<30 to >50	n.g.	untrained	no	n.g.	n.g.	no
11	Bouty-Regard et al. [27]	2020	m + w	77 ± 2	21.5	untrained	yes	0	97	no
12	Centin et al. [28]	2017	w	25–40	27.6 ^5^	untrained	no	n.g.	n.g.	n.g.
13	DiCagno et al. [29]	2023	m + w	72 ± 6	n.g.	untrained	yes	0	100	no
14	Dyaksa et al. [30]	2022	w	n.g.	n.g.	untrained	no	n.g.	n.g.	no
15	Ethem et al. [31]	2019	w	38 ± 5	23.7	untrained	no	n.g.	n.g.	no
16	Evangelista et al. [33]	2019	m + w	26 ± 4	25.2	moderate	no	16	95	no
17	Evangelista et al. [32]	2021	m	75 ± 7	n.g.	untrained	no	33	100	no
18	Fritzsche et al. [34]	2010	m + w	27–73	26.8	untrained	yes	0	n.g.	no
19	Ghannadi et al. [35]	2022	w	33 ± 6	27.3	untrained	no	15	80	no
20	Hamada et al. [36]	2023	m + w	20–69	21.4	untrained	yes	12	71	no
21	Homma et al. [37]	2022	m + w	79 ± 6	22.0	untrained	yes	29	100	no
22	Houdjijk et al. [38]	2022	m + w	45–75	31.8 ^6^	untrained	yes	0	95	no
23	Imaoka et al. [39]	2022	m + w	64 ± 7	24.2	untrained	yes	27	n.g.	no
24	Jee et al. [40]	2019	m	25 ± 2	22.0	untrained	no	5	100	no
25	Junger et al. [41]	2020	m + w	18–62	23.0	moderate	no	0	100	no
26	Kataoka et al. [42]	2019	m + w	83 ± 6	16.7	untrained	yes	25	n.g.	no
27	Kemmler et al. [44]	2010	w	65 ± 6	26.0	well	yes	0	98	no
28	Kemmler et al. [43]	2010	m	69 ± 3	28.1	untrained	yes	7	78	no
29	Kemmler et al. [47]	2013	w	75 ± 4	22.1	untrained	yes	16	79	no
30	Kemmler et al. [45]	2016	m	30–50	28.5	moderate	no	13	90	no
31	Kemmler et al. [46]	2016	w	77 ± 4	25.1	untrained	yes	10	89	no
32	Kemmler et al. [11]	2017	m	77 ± 5	26.1	moderate	yes	9	91	no
33	Kim et al. [48]	2020	w	71 ± 3	30.9	untrained	yes	13	n.g.	no
34	Kim et al. [49]	2021	m	24 ± 2	25.1	moderate	no	7	n.g.	no
35	Kiriscioglu et al. [50]	2019	w	34 ± 9	25.3 ^7^	moderate	no	0	95	no
36	Konrad et al. [51]	2020	m + w	56 ± 14	n.g.	untrained	yes	27	85	no
37	Ludwig et al. [52]	2019	m + w	25 ± 4	23.9	moderate	no	10	100	no
38	Lukashevich et al. [53]	2020	w	45–65	n.g.	untrained	yes	n.g.	n.g.	no
39	Matsumoto et al. [54]	2020	m + w	66 ± 6	24.0	untrained	yes	n.g.	n.g.	no
40	Matsuo et al. [55]	2022	m + w	77 ± 11	24.0	untrained	yes	6	94	no
41	Micke et al. [56]	2021	m + w	40–70	26.3	moderate	yes	9	92	no
42	Miyamoto et al. [57]	2016	m	22 ± 2	21.4	moderate	no	0	n.g.	no
43	Mori et al. [58]	2020	m	65 ± 13	n.g.	untrained	yes	n.g.	n.g.	n.g.
44	Müllerova et al. [59]	2022	w	63 ± 2	26.6	untrained	no	18	n.g.	n.g.
45	Nakamura et al. [60]	2019	m + w	76 ± 12	21.0	untrained	yes	55	100	no
46	Nakamura et al. [61]	2021	m + w	68 ± 15	21.4	untrained	yes	17	100	n.g.
47	Nejad et al. [62]	2021	w	60–70	28.2	untrained	no	n.g.	n.g.	n.g.
48	Noguchi et al. [63]	2017	m + w	69 ± 10	n.g.	untrained	yes	0		no
49	Nonoyama et al. [64]	2022	m + w	72–84	24.4	untrained	yes	n.g.	97	no
50	Ochiai et al. [65]	2018	m + w	60–90	n.g.	untrained	yes	0	n.g.	no
51	Özdal et al. [67]	2016	w	32 ± 8	24.5	untrained	no	0	n.g.	no
52	Öktem et al. [66]	2022	W	22–27	23.6	untrained	no	0	n.g.	no
53	Pano-Rodriguez et al. [68]	2020	w	61 ± 4	26.5	untrained	no	6	93	no
54	Park et al. [71]	2021	w	70 ± 4	27.5	untrained	yes	6	100	no
55	Park et al. [70]	2021	w	61–79	24.4	untrained	yes	7	92	no
56	Park et al. [69]	2021	w	20–40	25.0	untrained	no	8	100	no
57	Park et al. [72]	2023	W	≥65	25.4	untrained	No ^8^	3	97	no
58	Qin et al. [73]	2022	m	25 ± 4	24.0	well	no	15	100	no
59	Reljic et al. [74]	2022	m + w	≥18	37.2	moderate	yes	23	93	no
60	Ricci et al. [75]	2020	m + w	32–45	38.2	moderate	yes	0	100	no
61	Richter et al. [76]	2019	m + w	≥18	25.5	untrained	yes	19	88	0
62	Sadeghipour et al. [78]	2021	w	26 ± 2	21.7	well	no	0	n.g.	n.g.
63	Sadeghipour et al. [77]	2022	w	32 ± 5	27.8	moderate	no	n.g.	100	no
64	Sanchez-Infante et al. [79]	2020	w	40–60	25.5	moderate	no	0	100	no
65	Schink et al. [80]	2018	m + w	≥18	25.2	untrained	yes	40	87	no
66	Schink et al. [81]	2018	m + w	≥18	25.4	untrained	yes	59	77	no
67	Schwappacher et al. [82]	2020	m	≥18	28.0	untrained	yes	n.g.	88	no
68	Schwappacher et al. [82]	2020	m + w	≥18	26.8	untrained	yes	n.g.	85	no
69	Schwappacher et al. [83]	2021	m + w	>18	24.6	untrained	yes	n.g.	79	no
70	Silvestri et al. [84]	2023	m + w	43–81	24.3	untrained	yes	23	91	no
71	Song et al. [85]	2020	W	20–25 ^4^	26.1	n.g.	no	n.g.	n.g.	no
72	Stephan et al. [86]	2023	m + w	25–36	25.3	untrained	no	7	80	no
73	Struhar et al. [87]	2019	w	23 ± 2	23.2	untrained	no	n.g.	n.g.	n.g.
74	Suzuki et al. [88]	2018	m + w	65 ± 7	23.7	n.g.	yes	13	98	no
75	Suzuki. et al. [89]	2018	m + w	66 ± 10	26.7	untrained	yes	0	n.g.	no
76	Tanaka et al. [90]	2022	m + w	>75	21.6	untrained	yes	25	86	no
77	Teschler et al. [92]	2016	m	20–50	24.9	well	no	0	100	Yes ^9^
78	Teschler et al. [91]	2021	m + w	56 ± 7	35.7	moderate	yes	4	98	no
79	Tsurumi et al. [93]	2022	m + w	74 ± 5	22.7	untrained	yes	27	n.g.	n.g.
80	Vacoulikova et al. [94]	2021	w	60–65	27.1	untrained	no	n.g.	81	no
81	Vacoulikova et al. [95]	2021	w	60–65	27.0	untrained	yes	18	100	no
82	van Buuren et al. [97]	2014	m + w	61 ± 13	29.7	untrained	yes	0	100	no
83	van Buuren et al. [96]	2015	m + w	62 ± 3	34.6	untrained	yes	0	100	no
84	von Stengel et al. [98]	2015	w	>70	22.2	untrained	yes	16	79	no
85	Weissenfels et al. [99]	2018	m + w	57 ± 7	27.9	moderate	yes	7	93	no
52	Willert et al. [100]	2019	w	25–50	31.3	moderate	yes	3	100	no
67	Zink et al. [101]	2021	m	18–70	27.4	moderate	no	33	95	no

^1^: due to the approach of calculating BMI by body length and mass in case of missing BMI we do not list the SD here; ^2^: untrained: no regular exercise; moderate: 1 session per week, well: 2–3 sessions per week; ^3^ Drop-out rate of the WB-EMS group(s); ^4^: Blöckl et al. (2022): frail cohort; ^5^: Cetin et al. (2017): cohort 36–40 years old; ^6^ Houdjik et al. (2022): participants with non-insulin-dependent diabetes; ^7^ Kiriskoglu et al. (2019): WB-EMS group; CG: 29 kg/m^2^; ^8^ Park et al. (2023): Prefrail older women; ^9^ Teschler et al. (2016).: The aim of the study was to generate rhabdomyolysis.

## Data Availability

The datasets generated and/or analyzed during the current study are available from the corresponding author on reasonable request.

## Supplementary Materials

The following supporting information can be downloaded at: https://www.mdpi.com/article/10.3390/s24030972/s1, Table S1: Search strategies and their results; Table S2: Exercise and stimulation characteristics of the included studies.
