# Supplementary material for: Non-Athletic Cohorts Enrolled in Longitudinal Whole-Body Electromyostimulation Trials—An Evidence Map
Source: Sensors (Basel). 2024 Feb 2;24(3):972. doi: 10.3390/s24030972 (PMC10857049; doi:10.3390/s24030972)
Supplement: Supplementary file 1 [file sensors-24-00972-s001.zip › sensors-2810137-supplementary.pdf]

Supplement

Table S1: Search strategies and their results

**Database**	**Search date**	**Search terms**	**Number of hits**
Medline (PubMed)	6th March 2023	(WB-EMS[TIAB] OR "whole-body electromyostimulation"[TIAB] OR "whole body electromyostimulation"[TIAB] OR "whole-body electrostimulation"[TIAB] OR "whole body electrostimulation"[TIAB] OR electromyostimulation[TIAB] OR "EMS training"[TIAB] OR "EMS intervention"[TIAB] OR "EMS suit*"[TIAB] OR "EMS belt*"[TIAB] OR "B-SES"[TIAB] OR "belt electrode"[TIAB])	421
CENTRAL	6th March 2023	(WB-EMS OR "whole-body electromyostimulation" OR "whole body electromyostimulation" OR "whole-body electrostimulation" OR "whole body electrostimulation" OR electromyostimulation OR "EMS training" OR "EMS intervention" OR "EMS suit*" OR "EMS belt*" OR "B-SES" OR "belt electrode"): ti,ab,kw	248
CINAHL	6th March 2023	1. TI (WB-EMS OR "whole-body electromyostimulation" OR "whole body electromyostimulation" OR "whole-body electrostimulation" OR "whole body) electrostimulation" OR electromyostimulation OR "EMS training" OR "EMS intervention" OR "EMS suit*" OR "EMS belt*" OR "B-SES" OR "belt electrode"<br><br>2. AB (WB-EMS OR "whole-body electromyostimulation" OR "whole body electromyostimulation" OR "whole-body electrostimulation" OR "whole body) electrostimulation" OR electromyostimulation OR "EMS training" OR "EMS intervention" OR "EMS suit*" OR "EMS belt*" OR "B-SES" OR "belt electrode"<br><br>3. S1 OR S2	104
SPORTDiscus	6th March 2023	1. TI (WB-EMS OR "whole-body electromyostimulation" OR "whole body electromyostimulation" OR "whole-body electrostimulation" OR "whole body) electrostimulation" OR electromyostimulation OR "EMS training" OR "EMS intervention" OR "EMS suit*" OR "EMS belt*" OR "B-SES" OR "belt electrode"<br><br>2. AB (WB-EMS OR "whole-body electromyostimulation" OR "whole body electromyostimulation" OR "whole-body electrostimulation" OR "whole body) electrostimulation" OR electromyostimulation OR "EMS training" OR "EMS intervention" OR "EMS suit*" OR "EMS belt*" OR "B-SES" OR "belt electrode"<br><br>3. S1 OR S2	185
PEDro	6th March 2023	Abstract & Title: "whole-body electromyostimulation"<br><br>Method: clinical trial	34

Clinicaltrials.gov	6th March 2023	other terms: WB-EMS OR "whole-body electromyostimulation" OR "whole body electromyostimulation" OR "whole-body electrostimulation" OR "whole body electrostimulation" OR electromyostimulation OR "EMS suit*" OR "EMS belt*" OR "belt electrode*"	39
ICTRP	6th March 2023	Search: WB-EMS OR "whole-body electromyostimulation" OR "whole body electromyostimulation" OR "whole-body electrostimulation" OR "whole body electrostimulation" OR electromyostimulation OR "EMS suit*" OR "EMS belt*" OR "belt electrode*"	72
Google Scholar	6th March 2023	Advanced search: "whole body electromyostimulation" OR "WB EMS" OR "EMS suit" OR "belt electrode" with at least one of the words, anywhere in the article	1293

Table S2 Exercise and stimulation characteristics of the included studies.

	**Author**	**EMS-System**	**Isolated EMS ?**	**Active mode ?**	**Intervention length (months)**	**Sessions /week (n)**	**Session length (min)**	**Impulse frequency (Hz)**	**Impulse width (µs)**	**Impulse length (s)**	**Impulse break (s)**
1	Afsharnezhad et al.[17]	WB-EMS	yes	yes	2	3	90	30 to 85	n.g.	6	6
2	Akcay et al.[18]	WB-EMS	yes	yes	1	2	20	85,	350	30	10
3	Almada et al.[19]	WB-EMS	no	yes	1	2	20	n.g.	n.g.	n.g.	n.g.
4	Amaro-Gahete et al.[21]	WB-EMS	no	yes	1.5	1	12 - 20	12 to 90	350	4 - 30	4 - 30
5	Amaro-Gahete et al.[20]	WB-EMS	no	yes	3	2	20 or 32,5	15-20 and 35-75	200 - 400	6	4
6	Andre et al.[22]	WB-EMS	yes	yes	1.5	5	25	30 and 85	350	6	4
7	Bellia et al.[23]	WB-EMS	yes	yes	6	2	20	15 or 85	400	4[2]	4
8	Berger et al.[24]	WB-EMS	yes	yes	2.5	1.50	20	20 or 85	350	4	4
9	Blöckl et al.[25]	WB-EMS	yes	yes	2	1-1.5	20	85	350	6	4
10	Bostan et al.[26]	WB-EMS	yes	yes	1	2	25	85	350	4	4
11	Bouty-Regard et al.[27]	B-SES	yes	n.g.	3	2	20	20	250	5	2
12	Cetin et al.[28]	WB-EMS	yes	Yes	2	2	25	85	350	4	4
13	DiCagno et al.[29]	WB-EMS	yes	yes	3	2	20	7 or 85	350	4	4
14	Dyaksa et al.[30]	WB-EMS	yes	yes	1.5	2	25	n.g.	n.g.	n.g.	n.g.
15	Ethem et al.[31]	WB-EMS	yes	yes	1.5	2	25	7 and 85	350	variable	variable
16	Evangelista et al.[33]	WB-EMS	no	yes	2	2	20	85	350	1200	0
17	Evangelista et al.[32]	WB-EMS	no	yes	1.5	2	20	85	350	4	2
18	Fritzsche et al.[34]	WB-EMS	yes	yes	6	2	20	80	300	4	4
19	Ghannadi et al.[35]	WB-EMS	no	yes	1.5	2	20	85	350	6	4
20	Hamada et al.[36]	B-SES	yes	yes	1	7(?)	20	20	250	5	2
21	Homma et al.[37]	B-SES	yes	no	3	3	40	20	250	5	2
22	Houdjijk et al.[38]	WB-EMS	yes	yes	4	2	20	85	350	4	4
23	Imaoka et al.[39]	B-SES	yes	no	0.5	5	20	20	250	5	2
24	Jee et al.[40]	WB-EMS	yes	yes	1.5	3	20	85	350	6	4

25	Junger et al.[41]	WB-EMS	yes	yes	1	2	20	85	40	4	4
26	Kataoka et al.[42]	B-SES	yes	No	3	3	20	4	250	1200	
27	Kemmler et al.[44]	WB-EMS	yes	Yes	3.5	2	20	7 and 85	350	4	4
28	Kemmler et al.[43]	WB-EMS	yes	Yes	3.5	1.50	30	85	350	4	4
29	Kemmler et al.[47]	WB-EMS	yes	Yes	12	1.50	20	85	350	6	4
30	Kemmler et al.[45]	WB-EMS	yes	Yes	4	1.50	20	85	350	6	4
31	Kemmler et al.[46]	WB-EMS	yes	Yes	6	1	20	85	350	4	4
32	Kemmler et al.[11]	WB-EMS	yes	Yes	4	1.50	20	85	350	4	4
33	Kim et al.[48]	WB-EMS	no	Yes	2	3	40	85	350	6	4
34	Kim et al.[49]	WB-EMS	yes	Yes	3	3	20	85	350	6	4
35	Kiriscioglu et al.[50]	WB-EMS	yes	Yes	2	2	25	85	350	4	4
36	Konrad et al.[51]	WB-EMS	yes	Yes	1.5	1	20	85	350	4	4
37	Ludwig et al.[52]	WB-EMS	yes	Yes	2.5	1.50	20	20 or 85	350	4	4
38	Lukashevich et al.[53]	WB-EMS	no	Yes	0.66	4	20	Up to 25000	Up to 5000	n.g.	n.g.
39	Matsumoto et al.[54]	B-SES	yes	no	1	5	20	20	250	5	2
40	Matsuo et al.[55]	B-SES	yes	n.g.	0.5	5	20	20	250	5	2
41	Micke et al.[56]	WB-EMS	yes	Yes	3	1	20	85	350	6	4
42	Miyamoto et al.[57]	B-SES	yes	n.g.	1	4	30	4	250	1200	
43	Mori et al.[58]	B-SES	yes	n.g.	1.5	2	30	20	250	5	2
44	Müllerova et al.[59]	WB-EMS	yes	yes	2.5	1	20	85	350	4	4
45	Nakamura et al.[60]	B-SES	yes	n.g.	0.5	7	20	20	250	5	2
46	Nakamura et al.[61]	B-SES	yes	n.g.	0.5	7	20	20	250	5	2
47	Nejad et al.[62]	WB-EMS	no	yes	3	3	20	15-33 and 35-75	200 and 400	6	4
48	Noguchi et al.[63]	B-SES	yes	n.g.	3	3	20	20	250	5	2
49	Nonoyama et al.[64]	B-SES	yes	n.g.	1.30	5	30	20	250	5	2
50	Ochiai et al.[65]	B-SES	yes	n.g.	1.10	7	20	20	250	5	2
51	Özdal et al.[67]	WB-EMS	yes	no	2	3	25	80	350	4	4
52	Öktem et al. [66]	WB-EMS	yes	yes	1.5	3	20	85	250	6	2
53	Pano-Rodriguez et al.[68]	WB-EMS	no	yes	2.5	2	40	7 and 55	150 to 350	6	4
54	Park et al.[71]	WB-EMS	no	yes	1.5	3	40	80	n.g.	5	3
55	Park et al.[70]	WB-EMS	no	yes	2	3	20	85	350	6	4
56	Park et al.[69]	WB-EMS	yes	yes	1.5	3	20	80	n.g.	5	3
57	Park et al. [72]	WB-EMS	no	yes	2	3	45	4	n.g.	2	2
58	Qin et al.[73]	WB-EMS	no	yes	1.5	3	20	85	350	6	4
59	Reljic et al.[74]	WB-EMS	yes	yes	3	2	20	85	350	6	4
60	Ricci et al.[75]	WB-EMS	yes	yes	1.5	5	27	30 and 85	350	6	4
61	Richter et al.[76]	WB-EMS	Yes	Yes	3	2	20	85	350	6	4
62	Sadeghipour et al.[78]	WB-EMS	yes	yes	1.5	2	20	85	350	6	4
63	Sadeghipour et al.[77]	WB-EMS	yes	yes	2	2	20	7-15 and 85	350	4	4
64	Sanchez-Infante et al.[79]	WB-EMS	no	yes	2	1	20	10 and 85[2]	350	8/cont.	4/cont.
65	Schink et al.[80]	WB-EMS	yes	yes	3	2	20	85	350	6	4
66	Schink et al.[81]	WB-EMS	yes	yes	3	2	20	85	350	6	4
67	Schwappacher et al.[82]	WB-EMS	yes	yes	3	2	20	85	350	6	4

68	Schwappacher et al.[82]	WB-EMS	yes	yes	3	2	20	85	350	6	4
69	Schwappacher et al.[83]	WB-EMS	yes	yes	3	2	20	85	350	6	4
70	Silvestri et al.[84]	WB-EMS	yes	yes	2	2	20	85	350	6	4
71	Song et al.et al.[85]	WB-EMS	no	yes	1	3	20	60	n.g.	5	5
72	Stephan et al.[86]	WB-EMS	yes	no	1.5	1	20	85	350	4	4
73	Struhar et al.[87]	WB-EMS	yes	yes	2.5	1.50	22	100	350	4	15
74	Suzuki et al.[88]	B-SES	yes	n.g.	2	3	20	20	250	5	2
75	Suzuki. et al.[89]	B-SES	yes	no	3	3	30	20	250	5	2
76	Tanaka et al.[90]	B-SES	yes	no	0.30	5	35	20	250	5	2
77	Teschler et al.[92]	WB-EMS	yes	yes	2.5		20	85	350	6	4
78	Teschler et al.[91]	WB-EMS	yes	yes	1	1.50	20	85	350	4	4
79	Tsurumi et al.[93]	B-SES	yes	no	3	3	30	4	250	1200	0
80	Vacoulikova et al.[95]	WB-EMS	yes	yes	2.5	1	20	85	350	4	4
81	Vacoulikova et al.[94]	WB-EMS	yes	yes	2.5	1	20	85	350	4	4
82	van Buuren et al.[97]	WB-EMS	yes	yes	2.5	2	20	80	350	4	4
83	van Buuren 2015 et al.[96]	WB-EMS	yes	yes	2.5	2	20	80	350	4	4
84	von Stengel et al.[98]	WB-EMS	yes	yes	12	1.50	20	85	350	6	4
85	Weissenfels et al.[99]	WB-EMS	yes	yes	3	1	20	85	350	6	4
86	Willert et al.[100]	WB-EMS	no	yes	4	1.50	20	85	350	6	4
87	Zink et al.[101]	WB-EMS	yes	yes	4	1	20	85	350	6	4

[1] This study (Teschler et al. 2016}) intentionally applied a very intense WB-EMS protocol for novice applicants to determine the effects of WB-EMS on parameters related to rhabdomyolysis; [2] (Bellia et al. 2020) 15 min with intermittent impulse, 5 min with continuous impulse (15 Hz); [3] (Öktem et al.2022) 10 min with intermittent, 10 min with continuous impulse.
